# The role of light in Chagas disease infection risk in Colombia

**DOI:** 10.1186/s13071-015-1240-4

**Published:** 2016-01-05

**Authors:** Diana Erazo, Juan Cordovez

**Affiliations:** BIOMAC, Universidad de los Andes, Bogota, Colombia

**Keywords:** Mathematical modeling, Chagas disease, *Rhodnius prolixus*, Risk factors, House infestation, Domestic lights

## Abstract

**Background:**

Chagas disease is the most important vector-borne disease in Latin America and *Rhodnius prolixus* is the main vector in Colombia. Control strategies in this region have shown poor outcomes due to the insect’s ability to disperse between the sylvatic and the domestic habitat. Because insect migration to houses is responsible to sustain contact rates between vectors and humans, understanding the risk factors that promote migration could be important in designing control strategies. In this respect, it has been reported that adult triatomines have the ability to move over long ranges at night attracted by artificial light. Thus, light bulbs could be playing a critical role in house invasion. The main objective of this study is to understand the role of artificial light, or simply *light*, in house infestation by *R. prolixus*.

**Methods:**

To investigate the role of light, we combined fieldwork in the village of Chavinave, Casanare, Colombia and a mathematical model of *Rhodnius prolixus* dynamics. The model allowed us to simulate insect mobility and distribution in the village based on field results. We created 11 scenarios representing different amounts of *light* in the village (from 0 to 100 %, with increments of 10 %) with 100 simulations each for a time of 1000 days (2.7 years) and compare the results between the scenarios.

**Results:**

None of the Gomez-Nuñez traps were positive at any stage of the study, suggesting that insects do not colonize houses. The model predicts that with current village connections the proportion of houses that have visiting insects should be around 98 %. Additionally we showed that an increase in *light* allows for insect spreading and migration to previously un-infested areas.

**Conclusions:**

Increments in *light* could increase the contact rates between vectors and humans; a two-fold increase in human cases for a 30 % increase in the use and visibility of *light* on this particular village was estimated with the model.

## Background

Chagas disease is caused by the parasite *Trypanosoma cruzi* and is transmitted to humans by insects of the subfamily Triatominae (Hemiptera: Reduviidae). It is a major public health problem in Latin America and recent estimates suggest that 6–8,000 000 people are infected and 65,000 000 are at risk of contracting the disease [1]. In Colombia, it is estimated that 436,000 individuals are infected (1 % of the population) [2, 3]. The main vector in the country is *Rhodnius prolixus* [4], which is characterized by high susceptibility of infection with *T. cruzi* and high mobility between the sylvatic (palm trees) and domestic habitat [5].

House infestation control efforts have focused mainly on fumigation. In Colombia, however, spraying has been of limited success [6] because of a strong house re-infestation due to the vectors’ ability to move between houses and palms [7, 8]. It has been suggested that contact rates between insects and people are an important aspect for disease establishment [9]; thus, understanding the factors that alter and increase vector-human encounters could be important in designing new control strategies.

Unfortunately, the eco-epidemiology of the disease is complex and human infection could be determined by several risk factors. These fall in two main categories: i) factors related to house infestation and ii) factors related to insect migration from palm trees to human dwellings. House infestation has been studied relatively well; it has been reported that insects like hiding in houses with adobe walls, palm leaves roofs and unfinished floors [10–16]. In addition, people [16] and domestic animals [16, 17] provide meals for vectors in the domicile and become reservoirs for the parasite; therefore, their densities have also been suggested to contribute to insect domiciliation. On the other hand, migration could be promoted by factors like light bulbs and the distance between the sylvatic and the domestic habitat, but they are poorly understood [18–21].

Recent studies demonstrated that adult Triatomines have the capability to move long distances at night [22–26] and it has been widely reported that artificial light is a powerful attractor for them [18–20, 27–29]. Thus, in principle, insects respond to light stimuli and have the potential to travel far. In addition, Triatomines are most active and likely to disperse when the temperature decreases, (i.e. night time) [20, 21, 25, 30–33]. For *R. prolixus* and *Triatoma infestans,* studies have found that their feeding patterns and sensitivity to host signals peaks in the early evening [19, 32, 34, 35] making lights in the peridomicile and domicile attractive for them. These findings suggest that rather than randomly dispersing among habitats, Triatomines could use directed movement towards human settlings that are within reach at particular times of the day and could prefer those with incandescent lamps. For instance, palm juice containers lit at night and open to contamination with triatomines, have been identified as a risk for oral transmission [28, 36, 37].

Therefore, understanding the role that artificial light could play in house invasion can have important consequences for disease prevention [22, 33, 38–40]. But quantifying experimentally the role of artificial light in relation to house infestation by *R. prolixus* is a difficult task. The main challenge could be ethical considerations that impede setting up experiments within occupied dwellings, but also such an experiment would require a complicated and expensive system to track insect movement.

In this study we investigate the role of artificial light in the distribution and abundance of insects in a real village, and its possible consequences in terms of contact rates with *R. prolixus*. We collected data in the village of Chavinave, located in Mani (Casanare), where our group recently reported densities and natural infection with *R. prolixus* in native palms of *Attalea butyracea* [41]. We observed and recorded house and palm infestation and their geographical position. Then we coupled our observations with a mathematical model that accounts for insect population dynamics including migration in an explicit environment that captures the network of habitats for insects in the village. The model allowed us to compute insect distribution, densities and fluxes between habitats. The model predicts a two-fold increase in cases for a 30 % increase in the use and visibility of light on this particular village.

## Methods

### Field-work

The department of Casanare is known as an area of high-risk level of Chagas transmission [42] and reports suggest 100 % infestation indexes in palms and a 67 % natural infection with *T. cruzi* in *R. prolixus* [43]. Our work was developed in the village of Chavinave (43°39’24” N, 72°9’36” W), located in the endemic municipality of Mani (200 m.a.s.l.), which has an average temperature of 27 °C. See [41] for additional information about this region.

Two or three Gomez-Nuñez (1965) [44] boxes, depending on house characteristics, were placed in 30 dwellings from March to December 2012 to passively search for Triatomines. Boxes were inspected every three months and replaced if necessary. In addition, small plastic containers were left at every house to store insects fortuitously captured (i.e. insects attracted by light-bulbs). We collected geographical coordinates, number of inhabitants, construction materials and information about the presence of light bulbs for all 30 dwellings in the village.

Insects are also found in palm trees and in this region, *Attalea buttyracea* palms are ubiquitous and have high densities of insects [41, 45]. We found 71 palms within a 1 km radius measured from the village-limit and recorded their coordinates; palms were manually inspected for insects (results reported elsewhere [41]). In addition, we identified all the palms and houses from which one could see a particular house with a turned on light bulb at night.

According to the Act 134 of 2011 of the Ethical Committee for Research of the University of Los Andes, the project accomplished all scientific, technique and administrative rules for health research established in Resolution 008430 of 1993 of the Ministry of Health.

### Mathematical model

We constructed a mathematical model to simulate insect mobility and distribution in the village based on field study results. In the model we consider houses and palms from the Chavinave village to be habitats suitable for insect populations. We called these habitats “patches” and assumed that flying insects connect them. We included *R. prolixus* population dynamics within every patch (meta-populations) and migration of adults between patches. Insect dynamics are based on an age-structured population where *E*_*i*_(*t*), *N*_*i*_(*t*) and *A*_*i*_(*t*) denote number of eggs, nymphs and adult respectively in patch *i* at time *t*.

Thus, the rate of change of every age-group is described as follows:

1 $$ \frac{d{E}_i}{dt}=\lambda {A}_i-\frac{1}{\tau }{E}_i-{\delta}_E{E}_i $$

2 $$ \frac{d{N}_i}{dt}=\frac{1}{\tau }{E}_i\left(1-\frac{N_i+{A}_i}{K_i}\right)-\frac{1}{\gamma }{N}_i-{\delta}_N{N}_i $$

3 $$ \frac{d{A}_i}{dt}=\frac{1}{\gamma }{N}_i-{\delta}_A{A}_i-{\beta}_i{A}_i+{\displaystyle \sum_{j=1}^n{\alpha}_{ji}{\beta}_j{A}_j} $$

where *λ* is the per-female egg production, *τ*is the average hatching time, 1/*γ*the average nymph to adult maturity rate, *K*is a patch’s carrying capacity and, *δ*_*E*_, *δ*_*N*_ and *δ*_*A*_ are egg, nymph and adult per-capita mortality, respectively. Biological parameters with their values and units are summarized in Table 1.

**Table 1:** Model parameters. Most of the parameters correspond to reported biological and ecological characteristics of *R. prolixus* included in the model

Symbol	Name	Units	Value	Ref
*λ*	Birth rate	$\frac{individual}{day\cdot individual}$	1.3	[63]
*δ*_*E*_	Egg mortality rate	1/*day*	0.001	[63]
*δ*_*N*_	Nymph mortality rate	1/*day*	0.004	[64]
*δ*_*A*_	Adult mortality rate	1/*day*	Palms: 0.005 Houses: 0.05	[64] -
*τ*	Residency time from egg to nymph	*day*	15.4	[63]
*γ*	Residency time from nymph to adult	*day*	211	[63]
*K*_*i*_	Carrying capacity in patch *i*	*individual*	Palms: 20 Houses: 1 × 10^−3^	[35, 41, 45]
*σ*	Maximum per capita migration rate	$\frac{individual}{day\cdot individual}$	0.1	-
*η*	Number of individuals at which half of the maximum per capita migration rate occurs	*individual*	1 × 10^−6^	-
*L*_*ji*_	Presence of light at destiny patch *i* seen from patch *j*	*arbitrary units*	0,1	-
*D*_*ji*_	Euclidean distance between patch*j* and patch *i*	*meter*	-	-
	Maximum dispersal distance *R. prolixus*	*meter*	200	[46, 65]

*σ* and *η* (included in equation 4) were approximated using a simple assumption about the per-capita migration rate of insects in a patch, which also depends on the number of nymphs and adults. *L* is a matrix that represents which connections, with origin *j* and destiny *i*, have light at patch *i*. Each *L*_*ji*_ takes a binary value 0 (no light) or 1 (light). Accordingly, *D* is a matrix that represents the distance between connections and *D*_*ji*_ is the Euclidean distance in meters between patch *j* and *i*

Movement between patches is included in the model by the two last terms of equation 3. − *β*_*i*_*A*_*i*_ accounts for insects moving from patch *i* to any other patch and ${\displaystyle \sum_{j=1}^n{\alpha}_{ji}{\beta}_j{A}_j}$ accounts for insects leaving other patches and arriving at *i*. Both fluxes are modelled using a per-capita migration rate *β*_*i*_ assumed to have saturation kinetics described by the following first order hill-equation

4 $$ {\beta}_i=\frac{\sigma \left({N}_i+{A}_i\right)}{\eta +\left({N}_i+{A}_i\right)} $$

where *σ* is the maximum per-capita migration rate and *η* is the number of individuals at which half of the maximum per capita migration rate occurs.

Decision to fly from origin patch *j* to destination patch *i* is captured by the adjacency matrix *α*_*ji*_ where every entry is defined by:

5 $$ {\alpha}_{ji}=\left({L}_{ji}+1\right)\left(200-{D}_{ji}\right).\; if\;{D}_{ji}>200\; then\;{\alpha}_{ji}=0 $$

where *D*_*ji*_ is the Euclidean distance between patch *j* and patch *i,* and *L*_*ji*_ is a matrix with 1 at entry *j*, *i* if patch *i* has a light that can be seen from patch *j* and 0 otherwise*. L*_*ji*_ = 0 for every *j*when *i* is a palm (i.e. palms do not have light bulbs). Equation 5 assumes that the maximum distance an insect can overcome is 200 mts [46] and also it assumes that two patches that are at the same distance, one with light and the other without it, will make the former two times more attractive. The *α*_*ji*_ ' *s* for every *i* are normalized to produce an adjacency matrix with entries between 0 and 1, where 1 means maximum attractiveness.

### Model simulations and model output

The village contains 30 houses and 71 palms for a total of 101 patches. The system of 303 coupled differential equations was solved numerically using Matlab. All the runs started with the same initial conditions: 20 insects per palm [45] (5 eggs, 5 nymphs and 10 adults) and no insects in houses. The system was run until achieving steady state and saved all state variables and fluxes (approximately 1000 days or 2.7 years).

To investigate the role of light on insect distribution and movement we developed 11 scenarios that differ in the amount of links that are equal to 1 (*L*_*ji*_ = 1 in equation 5), from 0 to 100 %. In other words, we varied the proportion of links that connect houses with palms and houses with houses in the village from 0 % (total absence of light, *L*_*ji*_ = 0 for every *j*, *i* in equation 5) to 100 % (all houses have lights that can be seen from every other habitat, *L*_*ji*_ = 1 for every *j*, *i*). The scenarios were run 100 times and for each run we randomly assigned the entries in *L* that were equal to 1.

During each run we continuously monitored insect migration by computing four indices: First, we computed the proportion of infested houses (PIH) as the average number of houses visited by insects divided by the total number of houses at steady state. Second, because it is not only the percentage of houses visited but also the number of insects moving into houses that determines transmission risk, we calculated a visiting index (VI) as the average number of insects per house divided by the number of visited houses per unit of time. Finally, we recorded the flux of insects from palms to houses (FPH), houses to houses (FHH) and house to palms (FHP).

The village with all its patches can be regarded as a directed network where connection between patches are represented by bidirectional links that have their weights given by equation 5. Insects can move between patches that have link weights above zero. Because manipulating *L*_*ji*_ changes the network configuration (i.e. what is connected and how strong), for every simulation we computed three more indices that reflect network metrics: First, we computed the number of groups of connected nodes (e.g. number of clusters - NC). This measurement gives the number of disconnected units in the village; as more clusters appear, fragmentation increases. Second, we calculated the proportion of patches in the biggest cluster (PPBC) which gives a measurement of how many patches are disconnected from the main network [47, 48]. Third and finally, we calculated the average path length (APL) which gives a measure of integration and it is computed as the number of steps that it takes on average to link two random patches in the same cluster [47, 48].

## Results

### Field-work

**Fig. 1:**
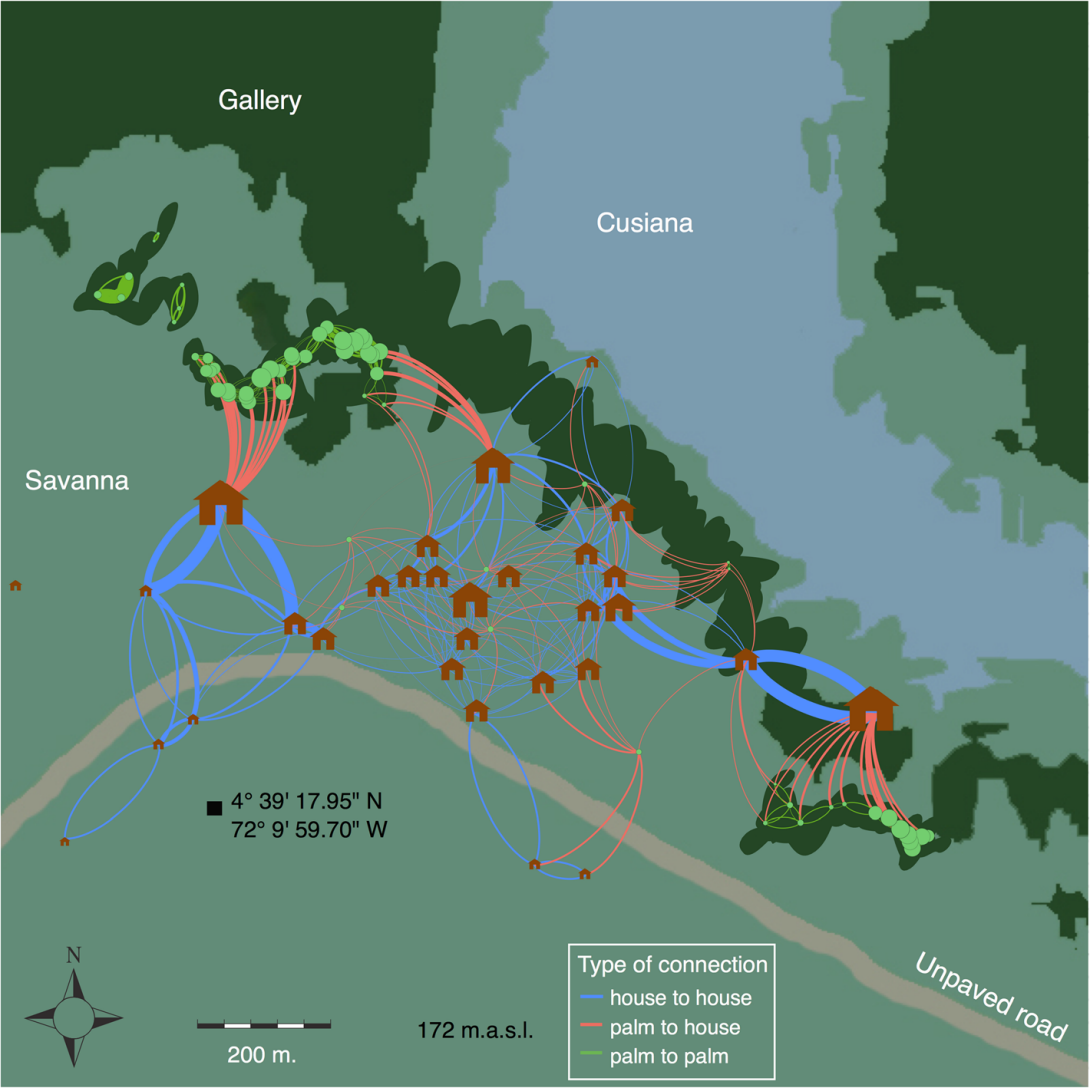
Study site. Chavinave is located in the municipality of Mani, Casanare (Colombia) and it has an area of 32 Ha. The village is adjacent to the Cusiana River (172 m.a.s.l.) and the landscape is characterized by Savanna and Gallery Forest. Houses and palms are shown at their exact location and are represented by the house and circle icons respectively. We developed a mathematical model that assumed houses and palms to be patches that contain meta-populations. The model consisted of an age structured population dynamics coupled with insect migration dependent on light distribution. The size of the patch is proportional to visiting index at every patch. The network shown in the figure summarizes average model output after 100 simulations run until steady state. Initial conditions assumed 20 insects per palm: 5 eggs, 5 nymphs and 10 adults and 0 insects per house. The simulation time was set to 1000 days (2.7 years)

Figure 1 shows a map of Chavinave with 30 houses and 71 palm trees. The village encompasses an area of approximately 32 ha. With the 101 patches we get a total of 3000 possible connections in the matrix *L* that can take the value of 1. When both distance (habitats that are close enough for insects to fly) and visibility (light is visible from source patch) are equal to 1, only 274 (64 %) connections remained present. 160 of those connections are from house to house and 114 are from palm to house. Out of the 7100 possible connections where *L* can be only 0 (or where there is no light involved because *j* is a palm), we found that 474 are within flying distance. Thus, patches are connected via two possible links: those that are based on proximity and those that are based on light and proximity. For Chavinave we had a total of 748 links, which is shown in Fig. 1.

The Gomez-Nunez traps [44] were inspected every 3 months for signs of eggs, nymphs or adults for all houses during the 9 months period, none of the traps were positive at any stage of the study. This result strongly suggests that insects do not colonize houses. However, residents reported seeing the adult insects come by at night supposedly attracted by lights several times. In 3 houses, where the owners were very diligent and agreed to capture insects that came attracted by the light bulb, 11, 3 and 1 adult *R. prolixus* were captured during a 1 week period and stored in plastic containers.

### Model simulations

**Fig. 2:**
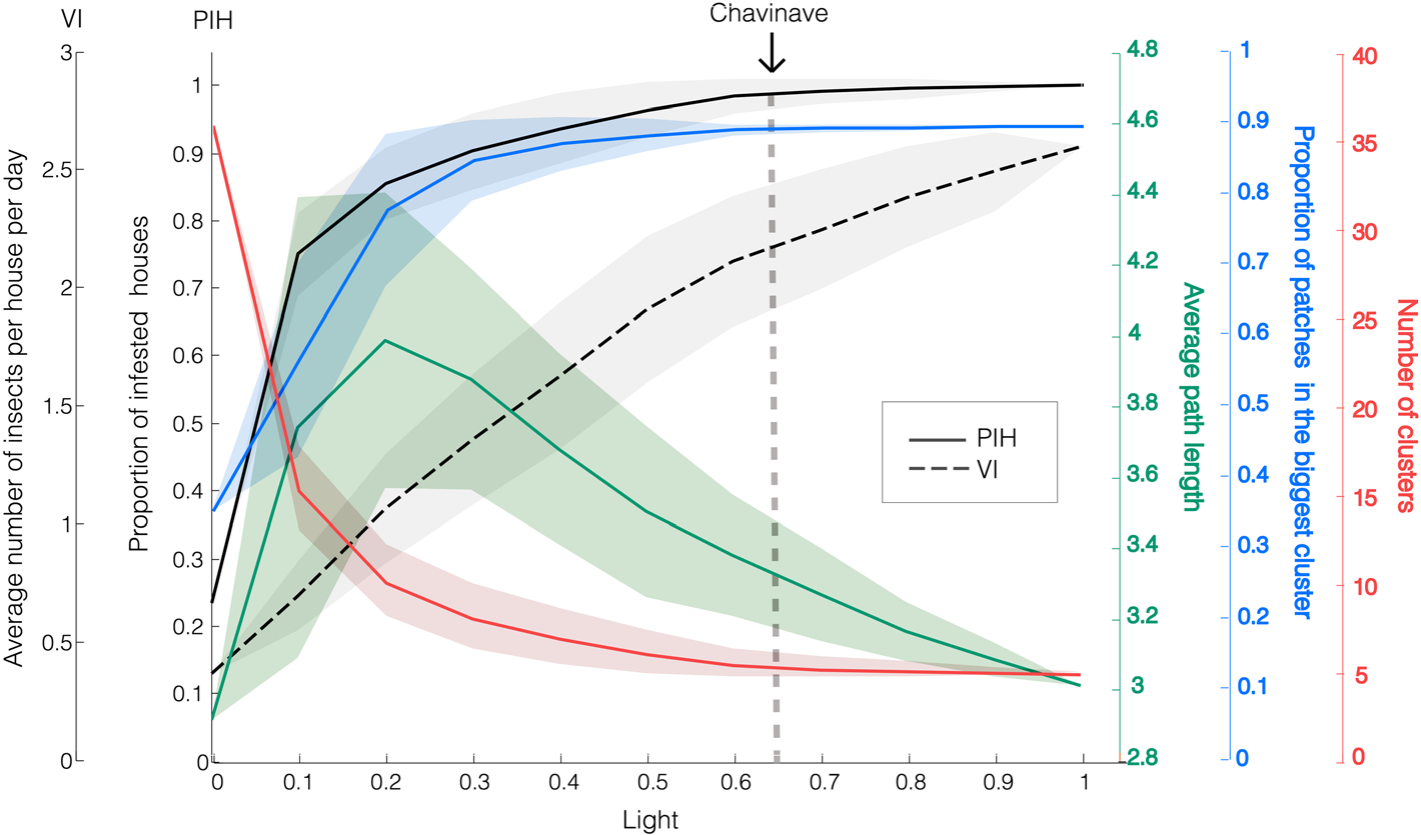
Epidemiological Indices I. Model outputs included: the average number of houses visited by insects divided by the total number of houses (proportion of infested houses - PIH) and the average number of insects per house per day or visiting index (VI). The mean and standard deviations at steady state for PIH and VI were calculated for every simulation while varying the proportion of patches that can actually see a house with light bulb at night (*light* on the *x axis*). Note that PIH exhibits a saturation effect. On the contrary, VI has a quasi-linear relation. In addition, for every *light* scenario we recorded network metrics: average path length (green), proportion of patches in the biggest cluster (blue) and number of clusters (red). We observed in the field that Chavinave has 274 connections out of 423 (64 %) possible. Thus, with the model we predict that Chavinave has a PIH of 0.98 and a VI of approximately 2 insects

**Fig. 3:**
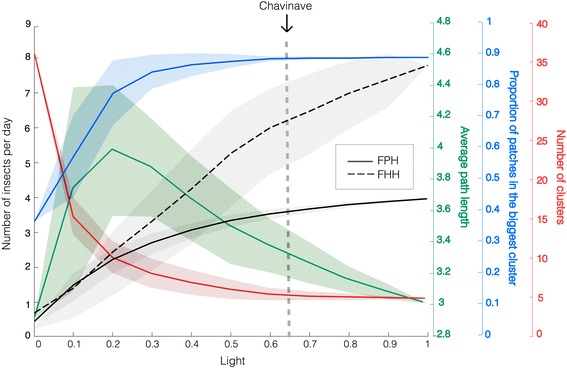
Epidemiological indexes II. Model output also included the average numbers of insects per day moving from palms to houses (FPH) and between houses (FHH) (*y* axis). Average fluxes and standard deviations were computed at steady state for every set of simulation for a particular value of *light* (*x* axis). Fluxes from houses to palms (FHP) were not observed in the simulations. Network metrics, as described in Fig. 2, are shown with solid colored lines with their corresponding standard deviations. For Chavinave the model predicts an FPH of 3.5 insects per day and an FHH of about 6.2 insects per day

**Fig. 4:**
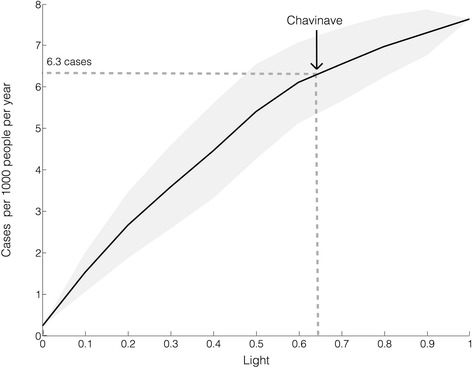
Predicted number of cases as function of light. The incidence, or number of new cases per person per year, was calculated based on the obtained VI, PIH, the proportion of infected insects [41], insect biting rate [51], feeding rate on humans [52] and probability of transmission per contact with an infected Triatomine [53]. For Chavinave, we estimate an incidence of 6.3 cases per 1000 people per year (see text for calculation). Using the current approximate population for Chavinave (122 people), the model predicts 1 new case every 18 months

We assumed that the proportion of entries in *L* that are equal to 1 reflects the presence of light in the village, and often in this manuscript we will refer to this variable simply as *light* (*x* axis in Figs. 2, 3 and 4). We did 11 breaks starting at *L*_*ji*_ = 0 for every *j*, *i* (0 %) to *L*_*ji*_ = 1 for every *j*, *i* (100 %). Because the entries equal to 1 were chosen randomly and repeated 100 times we could compute standard deviations, which are shown as shadows for every curve. Figures 2, 3 and 4 show hypothetical results for this village if we were to manipulate the network connectivity by varying entries in *L*. The current situation for Chavinave is described by the dotted line and corresponds to 64 %.

Figure 2 shows the relationship between VI and PIH with light and network metrics. As the number of times a house with a light bulb can be seen from another patch increases, PIH increases in a non-linear way with a saturation effect. Insects visit almost every house in the village when 50 % of more of the available connections are turned on. The model predicts that with current village connections (64 % of the entries in *L* are equal to 1) the proportion of houses that have visiting insects should be around 98 %. Figure 1 shows the connection between patches as well as the houses predicted by the model to have visiting insects. VI, on the other hand, has an approximate linear behavior. Interestingly, at 0 light, about 25 % of the houses with insects have an average of 0.3 insects per house per day (these connections are set by proximity alone). The number of insects increases to about 2.5 insects per house under the hypothetical condition that all patches are connected.

PPBC and NC show an opposite behavior: as the proportion of houses that have light increases the village has a higher PPBC while NC diminishes. As light goes to 100 %, NC is down to 5, but 90 % of the patches are all contained in one node. Thus, the village moves from 36 clusters at 0 % light to 5 where the village almost behaves like a single unit. We found that houses with the higher VI are all contained within the cluster that contains most of the nodes. In fact the other clusters are composed only by palm trees and one lonely house.

APL can determine whether a cluster is within reach for insects. At a low proportion of houses with light (0–10 %), APL is small because one has many clusters with small number of patches each. As the proportion of light increases, APL increases to a maximum of around 4 patches at 30 % light, and at this stage the number of clusters is close to the minimum but contains patches that are weakly connected. As light increases even further, APL decreases because patches increased their connection within roughly the same number of clusters. VI and PIH could be potentially linked to APL, however we found that the increase observed in APL is not at a level that impacts insect movement in the network.

FPH increases with a saturation behavior as the proportion of houses with light increases, reaching a maximum of 4 insects per day consistent with the connectivity created by light. On the other hand, FHH is always above FPH and shows a more linear behavior that reaches a maximum of 7.6 insects per day when all nodes are connected. This result is a consequence of the village organization: houses are close to each other with few palms within and the sylvatic habitat is in the periphery. At low light densities insects mostly see houses from palms but as lights start to increase the house network becomes predominant.

## Discussion

One of the most striking findings from this work was the fact that insects do not domiciliate in this small village in a highly endemic region. The paradigm for Chagas disease transmission in Colombia usually assumes insect domiciliation and the coexistence of a sylvatic and a domestic cycle [5, 45, 49, 50]. However, we could establish that insects often come to houses at night, around 7 pm, presumably attracted by artificial light [22–26] but do not seem to establish colonies. Thus, insects visit houses for a short period of time that could be meaningful for parasite transmission. We speculated that house-VI could be related to how visible and proximal was the house relative to palm trees. Therefore we evaluated the geographical position and the distance of every single house and palm tree in the village assuming they are the only possible habitats for Triatomines. In addition, we were able to establish which of the other habitats could see a light bulb that was hypothetically turned on at night for every house.

Because we wanted to explore the role of light in insect distribution and mobility, we developed a model that captured what we learned in the field coupled with published information about insect biology in order to account for population dynamics. Light is an attractor as long as the house is within reach and as such it is expected that light and geographical position have interacting effects. Thus, to investigate the role of light, it is important to understand that results highly depend on the configuration of the village. Incidentally, Chavinave is constructed such that all houses are grouped together at the center of the network and they are surrounded by Gallery Forest that contains palm trees. Caution must be taken when extrapolating the results from this study.

Before exploring the results of our model, is important to mention why house lights as opposed to streetlights are relevant for *R. prolixus* migration. *R. prolixus* tends to fly towards light in the early hours of the night, usually around 7 pm. This time coincides with human activities that involve use of light bulbs at home, like cooking or watching TV. In a recent study with *T. dimidiata,* researchers showed that this vector has peak activity between 1 and 4 am and thus it tends to infest the dwellings that are closer to public streetlights [33]. We did not consider public lights in our model.

The results obtained with this model suggest two interesting observations: i) light distribution and abundance affect VI and PIH because of a network effect and ii) once insects move to a house they do not return to the sylvatic habitat (i.e. palms); instead they stay circulating between houses due to a network effect. We explore these two interesting findings in some detail below.

The behavior of PIH and VI suggest that contact rates between insects and humans will increase as light increases. This is mainly due to an increase in VI because PIH is almost saturated at 50 %. This result suggests that every house in the village is exposed and the number of new cases will be dependent on the contacts between insects and humans. For the particular case of Chavinave we found that, on average, a house with a light bulb can be seen from 64 % of all available patches; at this level our model predicts a PIH of 98 % with a VI of approximately 2 visiting insects per house. We could not corroborate this result in the field because residents were not very diligent at capturing insects.

In theory, with this information, it is possible to estimate the number of human cases for Chavinave. First, we know from previous studies in the same area that the natural infection of Triatomines in palms is about 60.3 % [41]. Assuming that insects feed from humans once every 3–6 weeks [51], that *R. prolixus* feeds from humans half of the time (0.583 reported by [52]), and that the probability of transmission per contact with an infected Triatomine is about 5.8 × 10^−4^ [53], we obtain 3.9 × 10^−4^ new infected people per insect per year. Thus, for Chavinave, which has a human population of 122 people (in 2012) and an average of 2 insects per house per year, 98 % infested houses would result in 1 new case of Chagas every 18 months. Figure 4 shows the relationship between light and the number of cases following the same reasoning as above. This analysis predicts a two-fold increase in cases for a 30 % increase in light. Note that this analysis only focused light-related vectorial transmission and ignores oral transmission that could also increase due to light as suggested by several recent studies [28, 36, 37].

The second interesting finding relates to the observation that insects are trapped in the network of houses and do not return to the sylvatic environment. In other words we found that the flux from house to palms is 0. Another study showed that Triatomines that feed from humans in houses never return to the palm trees [54]. It is often believed that once an insect reaches the domestic cycle the next step is to colonize houses [55]. But here we find that insects would fly from house to house before returning to palms, which can have a tremendous impact on disease spreading but needs further investigation.

To understand this pattern of movement we can use network metrics. Evidently, light increments lead to a decrease in the number of clusters and an increase in the proportion of patches in the biggest cluster accompanied by a vast reduction in the average path length above 30 %. This is due to the fact that the biggest cluster has almost all the patches connected and the number of links between them increases with light presence. This situation allows for insect spreading and migration to new un-infested areas [56, 57]. In conclusion, insect movement is strongly influenced by the presence of light because light increases network connectivity. In addition the network behavior suggests that infestation risk for a particular house depends not only on its own light but also on its neighbors.

It is important to explore limitations of the model to highlight assumptions that naturally impose constrains to the results. First, we set insect migration to be entirely deterministic: insects choose the most attractive patch among a set of possible targets. This could be too simple as there might be some stochasticity involved. However, it has been suggested that Triatomines are strongly attracted by incandescent lamps [19, 28, 29] and adults could easily reach distant houses [22]. A second assumption is that light can make a patch twice as interesting as others located at the same distance and that the level of attractiveness decreases linearly with distance. This is clearly a simplification but we believe is a realistic starting point. For example, a recent study using a spatially explicit model shows that houses are from 5 to 15 times more attractive to bugs compared to the peridomicile and forest habitat [58]. Third, we assumed that all light bulbs were equally attractive, while literature suggests that white light bulbs are efficient in sampling insects [38], suggesting that wavelength is a key parameter [22]. Fourth, recent studies also suggest that seasonal patterns, which were not included in this model, can also affect insect migration [59]. Fifth, gender differences related to Triatomine movement have been reported that were not considered in our model. The dispersal of gravid females has been suggested to be relevant for the colonization of new habitats [25, 38]. Similarly, males fly less frequently but they do it for longer distances [60].

From a public health perspective, understanding how light shapes the movement patterns of insects in a village allows the development of precise risk maps [33, 58, 60]. In this study we show that houses near to palms are at much higher risk of infestation than houses further away, consistent with the experimental results reported by Ramirez-Sierra et al. (2010) [60] with *T. dimidiata* and modeled by Slimi et al. (2009) [61] . A risk map that considers not only the proximity to the sylvatic area [60] but also the possible connections among habitats where light is involved, will be more precise to determine possible *foci of transmission* in the village. This result can be used to spatially target houses for control strategies (i.e. insecticide spraying) [62]. Moreover, using unattractive light [33] or turning off target peridomicile lights in the village could be feasible vector control strategies.

## Conclusions

Our model coupled with the field work suggests: i) a two-fold increase in cases for a 30 % increase in the use and visibility of light on this particular village, ii) once populations of insects are trapped in the domestic environment they do not return to the sylvatic, iii) insect movement is strongly influenced by the presence of light because light increases network connectivity. In addition the network analysis showed that infestation risk for a particular house depends not only on its own status but also on its neighbor’s, and iv) developing risk maps that consider not only the proximity to the sylvatic area but also the possible connections among habitats, where light is involved, could help identify *foci* of transmission in the village and target specific houses for control strategies (i.e. insecticide spraying).

## Abbreviations

VI: Visiting index
PIH: Proportion of infested houses
FPH: Flux from palms to houses
FHP: Flux from houses to palms
FHH: Flux from houses to houses
FPP: Flux from palms to palms
PPBC: Proportion of patches in the biggest cluster
NC: Number of clusters
APL: Average path length
